# MHC-dependent inhibition of uterine NK cells impedes fetal growth and decidual vascular remodelling

**DOI:** 10.1038/ncomms4359

**Published:** 2014-02-28

**Authors:** Jens Kieckbusch, Louise M. Gaynor, Ashley Moffett, Francesco Colucci

**Affiliations:** 1 Department of Obstetrics and Gynaecology, University of Cambridge School of Clinical Medicine, NIHR Cambridge Biomedical Research Centre, Addenbrooke's Hospital, Box 111, Hills Road, Cambridge CB2 0SP, UK; 2 Centre for Trophoblast Research, University of Cambridge, Physiology Building, Downing Street, Cambridge CB2 3EG, UK; 3 Department of Pathology, University of Cambridge, Tennis Court Road, Cambridge CB2 1QP, UK

## Abstract

NK cells express variable receptors that engage polymorphic MHC class I molecules and regulate their function. Maternal NK cells accumulate at the maternal-fetal interface and can interact with MHC class I molecules from both parents. The relative contribution of the two sets of parental MHC molecules to uterine NK cell function is unknown. Here we show that, in mice, maternal and not paternal MHC educates uterine NK cells to mature and acquire functional competence. The presence of an additional MHC allele that binds more inhibitory than activating NK cell receptors results in suppressed NK cell function, compromised uterine arterial remodelling and reduced fetal growth. Notably, reduced fetal growth occurs irrespectively of the parental origin of the inhibitory MHC. This provides biological evidence for the impact of MHC-dependent NK inhibition as a risk factor for human pregnancy-related complications associated with impaired arterial remodelling.

## Abstract (web summary)

NK cells are involved in remodelling of the uterine vasculature during pregnancy and the extent of this process is influenced by the combination of maternal NK cell receptors and MHC-I of the fetus. Here, the authors provide further insights into how the presence of MHC-I from each parent differentially affects NK cell function.

Two key processes of placentation in both humans and mice are the transformation of the uterine spiral arteries supplying the developing placenta to ensure adequate feto-placental perfusion^1^,^2^ and the invasion of zygote-derived trophoblast cells into the decidua, which contains distinctive uterine natural killer cells (uNK). NK cells are innate lymphocytes that participate in both of these processes and are regulated by stress signals, adhesion molecules and receptors for MHC, including human killer-cell immunoglobulin-like receptors (KIR) and murine lectin-like Ly49 receptors, referred to herein as NKR^3^. Constant NKR-MHC interactions fine-tune NK responsiveness to match the MHC environment so that NK cells remain tolerant to self, yet responsive (a process termed education)^4^,^5^. MHC molecules can also influence NK maturation^6^ and directly inhibit or activate NK cell functions^3^. Both KIR and Ly49 are expressed in a variegated manner, such that individual NK cells express from zero to five NKR. This generates NK subsets with non-overlapping specificity, which express inhibitory or activating receptors that allow interaction with maternal (self) and paternal (allogeneic) MHC class I molecules, both of which are expressed by placental trophoblast cells^7^,^8^,^9^. The invasion of semi-allogeneic trophoblast cells into the decidua during pregnancy is a physiologically unique situation where maternal NK cells are directly exposed to novel, paternal MHC molecules^8^. A question that has not previously been addressed is whether paternal MHC contributes to uNK cell education by re-tuning their responsiveness and whether it is capable of modulating uNK function. Indeed, the exact role of the two sets of parental MHC in regulating maternal NK function is unknown, but, in human pregnancy, certain combinations of maternal NKR genes and fetal MHC genes predispose to complications of pregnancy such as pre-eclampsia, fetal growth restriction and recurrent miscarriage^7^,^10^,^11^. Pregnant women homozygous for the *KIR A* haplotype (characterized by predominantly inhibitory KIR) carrying a fetus bearing HLA-C2 are those most at risk, particularly when the fetal HLA-C2 is paternally derived^7^,^11^. This combination allows for strong inhibitory interactions between KIR2DL1 on maternal uNK and fetal HLA-C2. Conversely, the presence of the telomeric region of the *KIR B* haplotype, containing activating KIR2DS1 that can also bind HLA-C2, exerts a protective role^7^. This suggests that defective placentation and fetal growth may be caused by excessive inhibition of maternal uNK by paternal MHC, but direct experimental evidence for this is lacking. Moreover, uNK may develop in the uterus, where they could be educated to mature and acquire functional competence in response to signals from both sets of parental MHC.

Despite differences in murine and human pregnancy, uNK are a major lymphocyte population in the decidua of both species during placentation^12^,^13^,^14^. As in humans, invasive murine trophoblast cells express a unique MHC repertoire^8^, suggesting that NKR–MHC interactions regulate decidual functions similarly in both species^15^. The mouse may thus be an informative model in which to test the hypothesis that excessive inhibition of maternal uNK by paternal MHC class I molecules dampens their function and compromises reproductive fitness. We used a mating strategy in which *H-2D*^*d*^-expressing transgenic C57BL/6 (B6) mice (also known as D8)^16^ introduced an additional, allogeneic MHC class I gene that allows for stronger inhibition of uNK cells than in syngeneic B6 × B6 matings. By mating wild-type females with MHC transgenic males, we assessed the effect of paternal H-2D^d^ using multiple, complementary approaches. This mating strategy models combinations of maternal *KIR* and fetal *HLA-C*, where the introduction of an additional paternal MHC class I allele allows for strong inhibitory interactions between maternal NK cells and trophoblast cells. This mating strategy is also the only one in which the MHC of the fetus differs from that of the mother, and therefore the contribution of the paternal MHC class I transgene can be unambiguously determined. Here we show that subsets of uNK cells specific for the additional MHC class I allotype are less responsive. Functionally, this uNK inhibition is associated with compromised arterial remodelling and reduced growth in a large proportion of fetuses. Through matings where females are MHC transgenic (D8 × B6 and D8 × D8), we are also able to assess the functional consequences of maternal H-2D^d^ binding to inhibitory NKR on uNK cells during their development, rather than following acute exposure to it during pregnancy. In these matings, maternal uNK are likely to have been educated by the inhibitory NKR-MHC interactions before pregnancy. We show that fetal growth and arterial remodelling are impeded also in these matings. These findings suggest that MHC molecules derived from both parents can inhibit uNK expressing cognate receptors for that MHC class I allotype, irrespective of whether parental MHC are ‘matched’. Through genetic ablation of lymphocyte subsets, we demonstrate that NK cells are both necessary and sufficient to mediate these MHC-dependent effects on uterine vasculature and on fetal growth.

## Results

### Paternal MHC regulates uNK responsiveness

**Figure 1:**
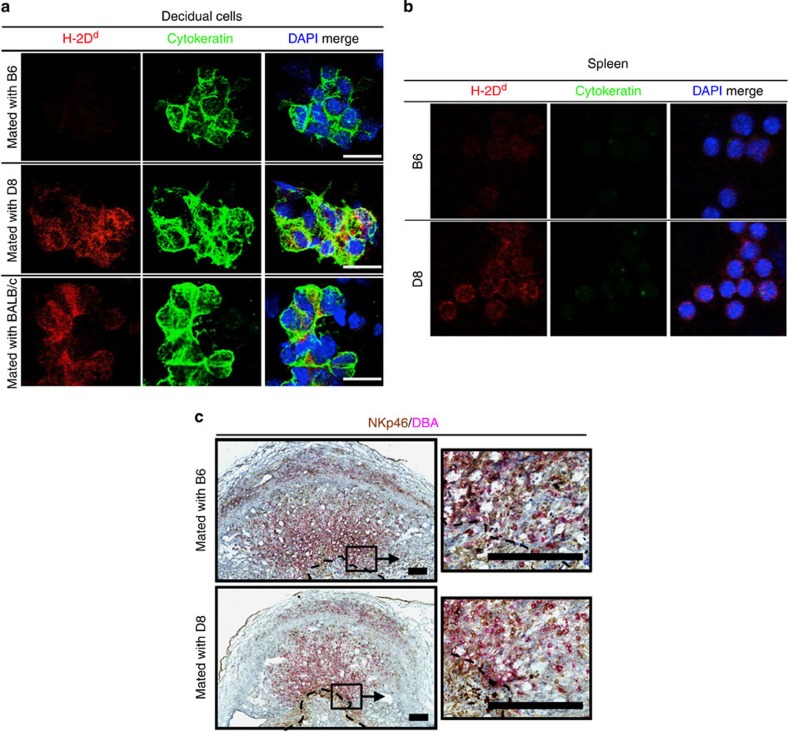
Paternal MHC expression on trophoblast. (**a**) Expression of paternal H-2D^d^ on gd9.5 trophoblast from B6 females impregnated by either B6 (top), D8 (middle) or BALB/c (bottom) males. BALB/c mice endogenously express H-2D^d^. (**b**) H-2D^d^ and cytokeratin staining on control spleen cells. Scale bar=25 μm. (**c**) Representative sections of the distribution of NKp46^+^ DBA^−^(brown) and NKp46^+^ DBA^+^ (pink) uNK in the region of trophoblast invasion in the central decidua at gd9.5. Dashed lines demarcate the approximate boundary between invasive ectoplacental cone trophoblast and maternal decidua basalis. Scale bar=250 μm. DBA, *Dolichos biflorus* agglutinin.

We first studied the B6 × D8 mating, in which we could assess the contribution of a paternally inherited and inhibitory MHC allotype exclusively expressed during pregnancy by the invading trophoblast. Trophoblast cells from B6 mice selectively express H-2K^b^, but not H-2D^b^ or non-classical MHC genes^8^. The pattern of MHC expression in other mouse strains, including the D8 transgenic mouse, is unknown. We therefore made cytospin preparations of decidual cell isolates from the B6 × D8 mating and stained them for both H-2D^d^ and cytokeratin to identify trophoblast cells that had invaded into the decidua. Indeed, both (B6 × D8) F1 and (B6 × BALB/c) F1 trophoblast cells as well as control spleen cells from D8 mice displayed expression of H-2D^d^ (Fig. 1a,b). Two uNK subsets have been described in the mouse, based upon their reactivity to *Dolichos biflorus* agglutinin (DBA)^17^. We were able to demonstrate that both subsets, marked by NKp46 and DBA, were in close proximity to trophoblast in the decidua (Fig. 1c).

The expression of H-2D^d^ by trophoblast in D8-mated B6 females allows for engagement of a greater number of inhibitory Ly49 than in B6-mated B6 females (Supplementary Fig. 1). To determine whether the presence of the additional trophoblast MHC class I molecule results in stronger inhibition of uNK, we measured intracellular IFN-γ, a cytokine predominantly produced by uNK and indispensible for complete uterine arterial remodelling in mice^12^,^17^,^18^,^19^,^20^,^21^. This assay of uNK responsiveness was performed by stimulating uNK *ex vivo* with IL-12 and IL-15, both of which are abundant in the decidua and important for uNK function^14^,^22^ (Supplementary Fig. 2). uNK from B6 females mated with either D8 or B6 males were compared. We focussed on IFN-γ producing NKp46^+^ DBA^−^ uNK cells^12^,^17^,^23^.

**Figure 2:**
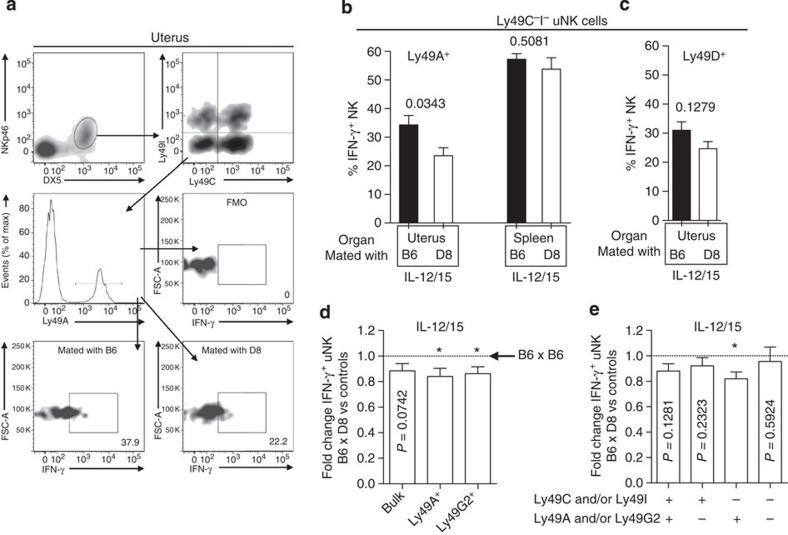
Paternal MHC regulates uNK responsiveness. (**a**) Gating strategy for Ly49C^−^I^−^Ly49A^+^ uNK (gd10.5) after exclusion of dead cells and CD3^+^ T cells. FMO, fluorescence minus one. (**b**,**c**) Frequency of IFN-γ^+^ NK cells (Ly49C^−^I^−^as gated in (**a**)) in response to IL-12 and IL-15 among Ly49A^+^ (**b**) or Ly49D^+^ (**c**). (**d**) Responsiveness of bulk NK cells and those expressing Ly49A or Ly49G2 (irrespective of expression of other Ly49) from B6 females mated with D8 males normalized to the responsiveness of uNK cells from B6 females mated with B6 males. (**e**) Responsiveness of uNK subsets with or without receptors recognizing H-2D^d^, *n*=5–8 mice per group. Means±s.e.m. *P-*values from unpaired Student’s *t*-tests, *P<0.05.

To resolve the relative contribution of paternal H-2D^d^, uNK expressing Ly49C and Ly49I were excluded, as these inhibitory NKR can bind maternal self-MHC in both matings^24^ (Supplementary Fig. 1). In contrast, inhibitory Ly49A has high affinity for H-2D^d^ but not H-2^b^ (ref. 24) and will inhibit uNK through paternally derived MHC molecules only in D8-mated B6 females. Two other low affinity NKR can also bind H-2D^d^ and have a ligand only in D8-mated B6 females: one inhibitory, Ly49G2 (ref. 24)^24^, and one activating, Ly49D^25^,^26^. Among maternal Ly49A^+^ uNK, but not splenic NK cells, fewer cells produced IFN-γ in D8-mated (23.5±2.8%) compared with B6-mated B6 females (34.3±3.3%) (Fig. 2a,b). Ly49D^+^ NK cells displayed similar IFN-γ production in both B6- and D8-mated B6 females, suggesting no impact of this activating Ly49 on uNK responsiveness in this mating combination (Fig. 2c). While IFN-γ production was similar in bulk uNK from D8-mated and B6-mated B6 females, both Ly49A^+^ uNK, comprising 10–15% of all uNK, and Ly49G2^+^ uNK, comprising 40–50% of all uNK (Supplementary Fig. 3), showed significantly lower IFN-γ production (Fig. 2d). Overall, uNK responsiveness was low when Ly49 specific for maternal MHC (Ly49C and Ly49I) were absent, while those specific for paternal MHC (Ly49A and Ly49G2) were present on the same cell (Fig. 2e, Supplementary Fig. 1). uNK responsiveness remained similar between the crosses in cells not expressing Ly49C/I/A/G2 (Fig. 2e). Thus, maternal uNK cell subsets that express inhibitory NKR specific for fetal MHC are less responsive.

### Maternal but not paternal MHC educates uNK cells

**Figure 3:**
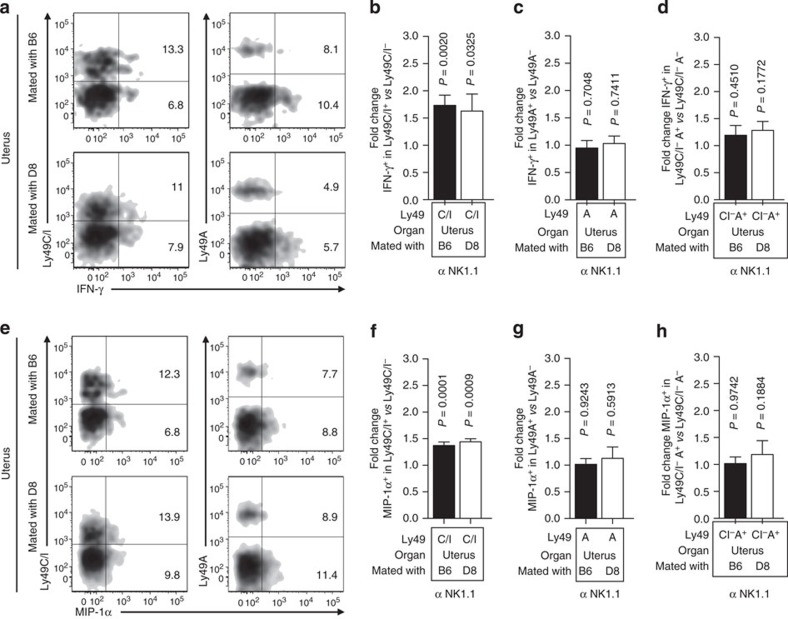
Paternal MHC does not educate uNK cells. (**a**) Density plots showing the proportion of IFN-γ producing uNK (gd9.5+10.5) in response to NK1.1 cross-linking among cells positive or negative for the indicated Ly49 (irrespective of expression of other Ly49). (**b**–**d**) Fold change in IFN-γ producing cells among uNK expressing the indicated Ly49 compared with cells that do not. Ly49C/I (**b**), Ly49A (**c**) and Ly49A after exclusion of Ly49C/I^+^ cells (**d**). (**e**) Density plots showing MIP-1α producing uNK cells (gd9.5+10.5) in response to NK1.1 cross-linking among cells positive or negative for the indicated Ly49 (irrespective of expression of other Ly49). (**f**–**h**) Fold-change in MIP-1α producing cells among uNK expressing the indicated Ly49 compared with cells that do not. Ly49C/I (**f**), Ly49A (**g**) and Ly49A after exclusion of Ly49C/I^+^ cells (**h**). Pooled data from two experiments, *n*=6–11 mice per group. *P*-values comparing uNK subset expressing a given Ly49 with those that do not within each mouse using paired Student’s *t*-tests. Means±s.e.m.

We next investigated the impact of paternal MHC on uNK education, which, in peripheral NK cells, is mediated by inhibitory NKR^4^,^5^. H-2K^b^ and H-2D^d^ educate Ly49C/I^+^ and Ly49A^+^ NK cells, respectively, such that the responsiveness of these specific NK subsets to subsequent ligation of activating receptors is enhanced. The proportion of educated cells is typically assessed using assays in which naive NK cells are stimulated *ex vivo* with antibodies that cross-link activating receptors (for example, anti-NK1.1) and resultant effector functions including cytokine production (IFN-γ, macrophage inflammatory protein (MIP)-1α) or degranulation (CD107a expression) measured. Educated NK cells expressing inhibitory receptors that bind self-MHC are expected to produce more IFN-γ than uneducated NK cells, which do not express receptors that bind to self-MHC^4^,^5^. The contribution of fetal MHC to education of uNK cells is unknown. Using this simple assay in our setting, the impact of maternal and paternal MHC molecules on uNK education can be quantified by measuring the frequency of IFN-γ producing cells within Ly49C/I^+^ (compared with ‘uneducated’ Ly49C/I^−^) and Ly49A^+^ (compared with ‘uneducated’ Ly49A^−^), respectively. The mean proportion of IFNγ-producing cells among Ly49C/I^+^ uNK was more than 1.5-fold higher than among Ly49C/I^−^uNK in both B6- and D8-mated B6 females, indicating that maternal MHC educates uNK cells. In contrast, there was no education from paternal MHC as Ly49A^+^ and Ly49A^−^cells were equally responsive in D8-mated B6 females (Fig. 3a–c). To exclude possible confounding effects of NKR binding to maternal MHC, we also assessed education in those cells that do not express H-2^b^-specific Ly49C or Ly49I and found that, also in this subset, Ly49A^+^ and Ly49A^−^cells were equally responsive in D8-mated B6 females (Fig. 3d), confirming that paternal H-2D^d^ does not educate uNK. Similar observations were made for MIP-1α production, thereby substantiating the finding that paternal MHC on fetal trophoblast does not educate maternal uNK (Fig. 3e–h).

### Paternal MHC on trophoblast does not affect uNK maturation

**Figure 4:**
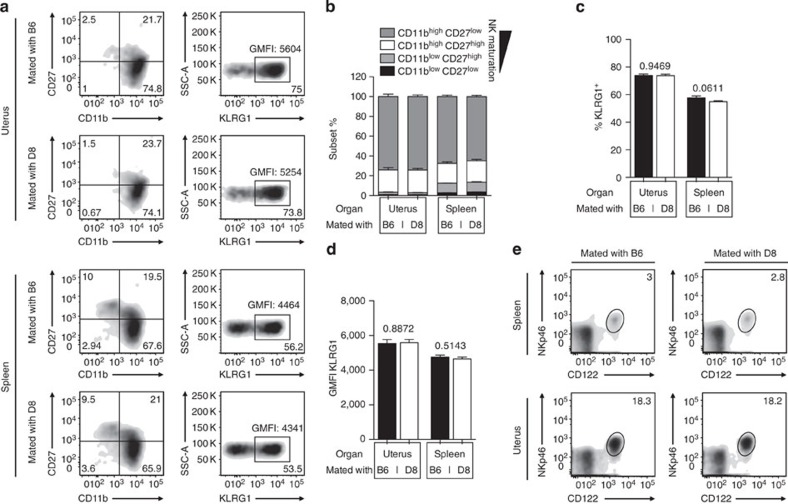
Paternal MHC does not influence uNK cell maturation. (**a**) Representative density plots showing the maturation of uNK (top 2 rows) and splenic NK cells (bottom 2 rows). (**b**,**c**) Frequency of NK cells in subsets corresponding to stages in maturation. (**d**) Geometric mean fluorescence intensity (GMFI) of KLRG1 expression in NK cells. Pooled data from three experiments, *n*=4–6 mice per group. Means±s.e.m. *P-*values from unpaired Student’s *t*-tests. (**e**) Representative density plots showing the frequency of CD122^+^ NKp46^+^ NK cells (gated on CD3^−^cells with lymphocyte size and granularity) in spleen and uterus.

The MHC environment can influence NK-cell maturation^6^ and we next asked whether the additional paternal MHC affected the maturational status of maternal NK cells. uNK in both matings contained more terminally mature CD11b^+^CD27^−^cells, suggesting that, despite their resemblance to peripheral NK cells, DBA^−^ uNK cells have a more differentiated phenotype. However, paternal H-2D^d^ did not affect the stage of NK maturation or expression of killer cell lectin-like receptor G1 (KLRG1) or their relative abundance, which were both comparable in B6- and D8-mated B6 females (Fig. 4a–e). Thus, paternal H-2D^d^ does not affect NK cell maturation. The relative size of Ly49 subsets was also similar, independent of the MHC of the male that had impregnated the female (Supplementary Fig. 3). Compared with splenic NK cells, uNK displayed an Ly49 repertoire skewed towards recognition of maternal MHC (Supplementary Fig. 3).

### Impaired arterial remodelling and myometrial abnormalities

**Figure 5:**
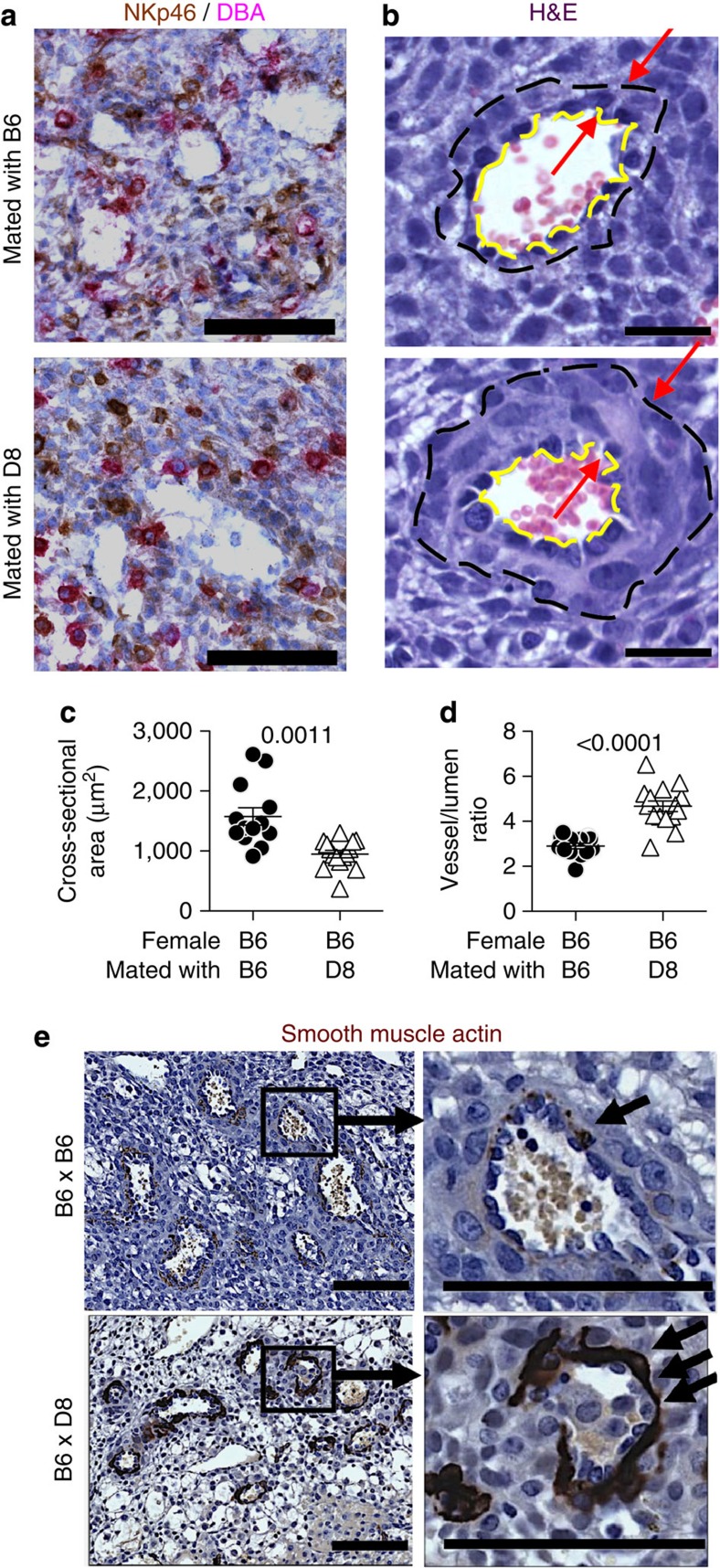
Paternal H-2D^d^ expression on trophoblast results in impaired arterial remodelling. (**a**) Immunohistochemical staining for NKp46 and DBA reveals abundant uNK around decidual vessels, independent of paternal MHC. Scale bar=100 μm. DBA, *Dolichos biflorus* agglutinin. (**b**) H&E staining of uterine arteries illustrating the strategy used to measure vessel size (yellow dashed ring) and relative thickness (surface area ratio between areas surrounded by yellow and black ring, red arrows). Scale bar=25 μm. (**c**–**e**) Stereological and immunohistochemical analysis of arterial size (**c**) and relative thickness (**d**) in the decidua of B6 females mated with either B6 or D8 males. Pooled data from 4 litters per cross, *n*=13–15 implantation sites. *P*-values from unpaired Student’s *t*-tests. Means±s.e.m. (**e**) Representative staining for smooth muscle actin showing that the characteristic loss of actin in the media of spiral arteries is reduced in matings with D8 males (black arrows). Scale bars=100 μm.

**Figure 6:**
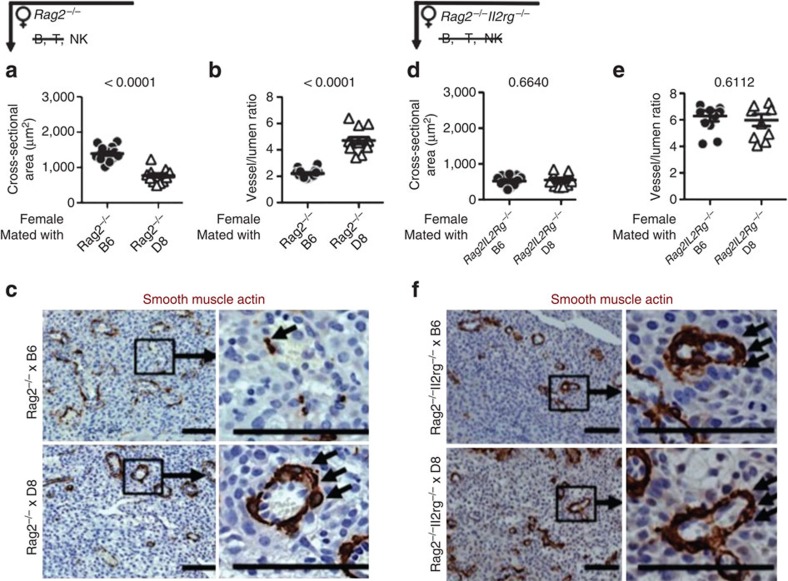
NK cells are necessary and sufficient for MHC-dependent impaired remodelling. (**a**–**c**) Stereological and immunohistochemical analysis of arterial size (**a**) and relative thickness (**b**) of B6.*Rag2*^−/−^ females (lacking mature T and B cells) mated with either B6 or D8 males. Pooled data from 4 litters per cross, *n*=12–14 implantation sites. *P*-values from unpaired Student’s *t*-tests. Means±s.e.m. (**c**) Smooth muscle actin staining showing that loss of actin in the media is reduced in matings with D8 males regardless of the absence of mature T and B cells (black arrows). (**d**–**f**) Stereological and immunohistochemical analysis of arterial size (**d**) and relative thickness (**e**) of B6*.Rag2*^−/−^*IL2rg*^−/−^ females (lacking mature NK, T and B cells) mated with either B6 or D8 males. Pooled data from 4 litters per cross, *n*=10 implantation sites. *P*-values from unpaired Student’s *t*-tests. Means±s.e.m. (**f**) Smooth muscle actin staining (black arrows) showing no MHC-dependent difference in the reduction of smooth muscle actin in the absence of mature NK, T and B cells (black arrows). Scale bars=100 μm.

Spiral artery remodelling is a hallmark of uNK function during pregnancy and has been reported to be compromised in female mice genetically modified to lack NK cells^12^,^14^,^18^,^27^. To assess how reduced responsiveness of uNK subsets caused by the presence of H-2D^d^ on trophoblast might affect the decidual vascular remodelling, a combination of stereology and immunohistochemistry was used. uNK were abundant around the vessels in B6 females regardless of the MHC status of the male they had been mated with (Fig. 5a). Arteries in the decidua of D8-mated B6 females had reduced luminal areas and thicker vascular walls compared with B6-mated B6 females (Fig. 5b–d), suggesting that strong MHC-dependent NK cell inhibition impedes arterial remodelling. Normally, transformation of arteries is associated with loss of smooth muscle actin in the vessel walls, and this was less pronounced in D8-mated B6 females (Fig. 5e). To exclude any contribution of other lymphocytes that might recognize allogeneic H-2D^d^, we used B6 female mice that lack functional T and B cells (B6.*Rag2*^−/−^) (ref. 28) and found that NK cells alone are sufficient to cause this phenotype (Fig. 6a–c). As an additional control, we assessed arterial remodelling in B6.*Rag2*^−/−^*II2rg*^−/−^mice, which are completely void of functional lymphocytes (B, T and NK cells)^29^. As previously reported^12^, we found overall reduced luminal areas, thicker vascular walls and abundant vascular smooth muscle actin in the decidual arteries in this NK-deficient strain compared with NK sufficient females, but no differences when comparing B6.*Rag2*^−/−^*II2rg*^−/−^females mated either with B6 or D8 males (Fig. 6d–f). These findings highlight the role of H-2D^d^ recognition by uNK to generate the MHC-dependent phenotype seen in D8-mated B6 females. Similarly, in the mesometrium of B6 females, where uNK are absent, arteries retained their normal structure regardless of the MHC of the male that had impregnated the female (Supplementary Fig. 4), further emphasizing the role of uNK cells in vascular remodelling. Taken together, these results show that NK cells are both necessary and sufficient for the MHC-dependent phenotype.

**Figure 7:**
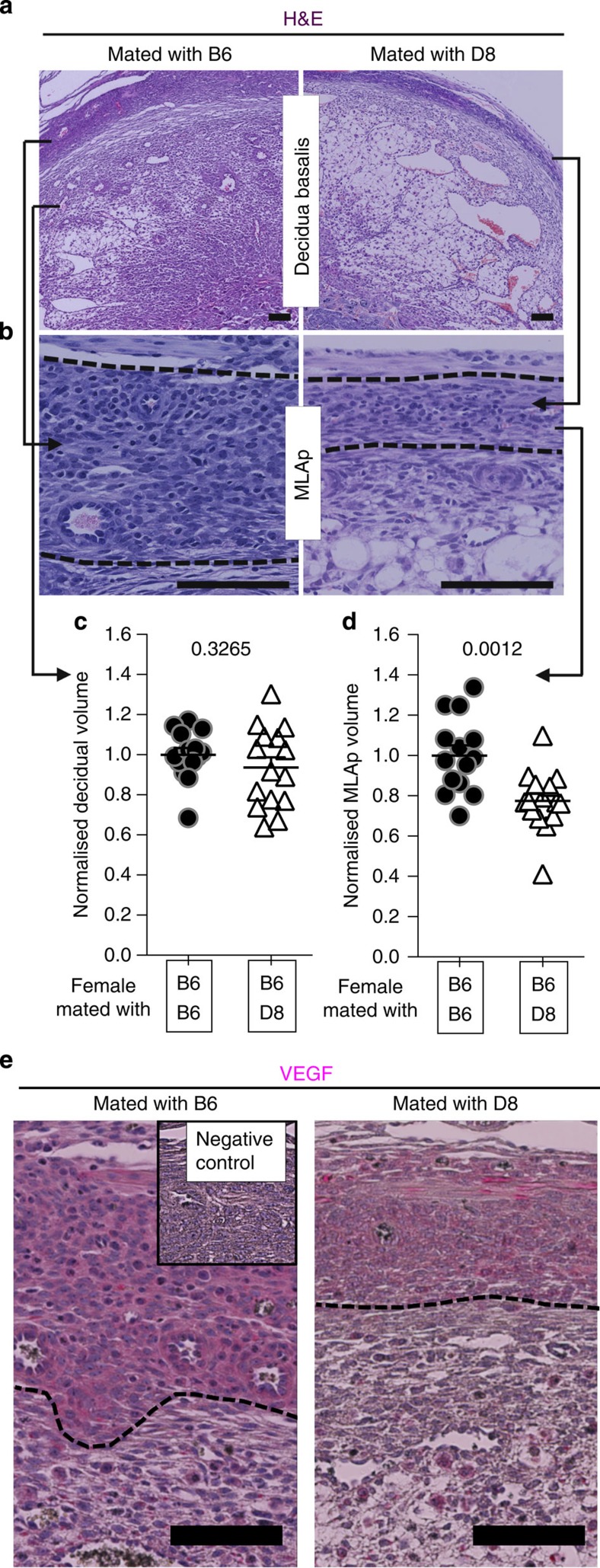
Myometrial abnormalities. (**a**,**b**) Representative sections of the pregnant uterus from B6 females stained with haematoxilin and eosin (H&E) illustrating the size of the decidua basalis (**a**) and MLAp (**b**). Dashed lines demarcate the smooth muscle layers that define the borders of the MLAp towards decidua and uterine wall. Scale bars=100 μm. (**c**,**d**) Stereological evaluation of decidual (**c**) and MLAp (**d**) volumes. Pooled data from 4 litters per cross, *n*=13–15 implantation sites. *P*-values from unpaired Student’s *t*-tests. Means±s.e.m. *P-*values from unpaired Student’s *t*-tests. (**e**) Representative sections of expression of VEGF-A and corresponding negative control in the decidua and MLAp of B6- and D8-mated B6 females. Scale bars=250 μm. H&E, haematoxylin and eosin. MLAp, mesometrial lymphoid aggregate of pregnancy. VEGF, vascular endothelial growth factor.

We next assessed decidualization and myometrial integrity in B6 females mated with either B6 or with D8 males. While the decidua was comparable in size in each conceptus, the mesometrial lymphoid aggregate of pregnancy (MLAp) was significantly smaller in D8-mated B6 females (Fig. 7a–d). The MLAp is a transient, lymphoid-like, myometrial structure of undefined function found in rodent pregnancies. It is located in the mesometrial pole and the main uterine arteries supplying the placenta coil through it. The MLAp contains large numbers of uNK and its size is severely reduced in NK-deficient mice^12^. We confirmed that cells within the MLAp, including uNK, produce pro-angiogenic vascular endothelial growth factor (VEGF)^30^,^31^, which is also produced by human uNK^13^ (Fig. 7e). It is likely that the significant reduction of MLAp volume in D8-mated B6 females negatively impacts on VEGF production in this region, which may have downstream functional consequences on decidual neovascularization, with possible effects on vascular changes in the decidua (Fig. 5b). uNK have been reported to influence trophoblast migration both positively, through chemoattraction^13^, and negatively, by limiting invasion^32^. Assessment of depth of the implanting placenta revealed no significant differences in trophoblast invasion between B6- and D8-mated B6 females (Supplementary Fig. 5).

### Reduced fetal growth

**Figure 8:**
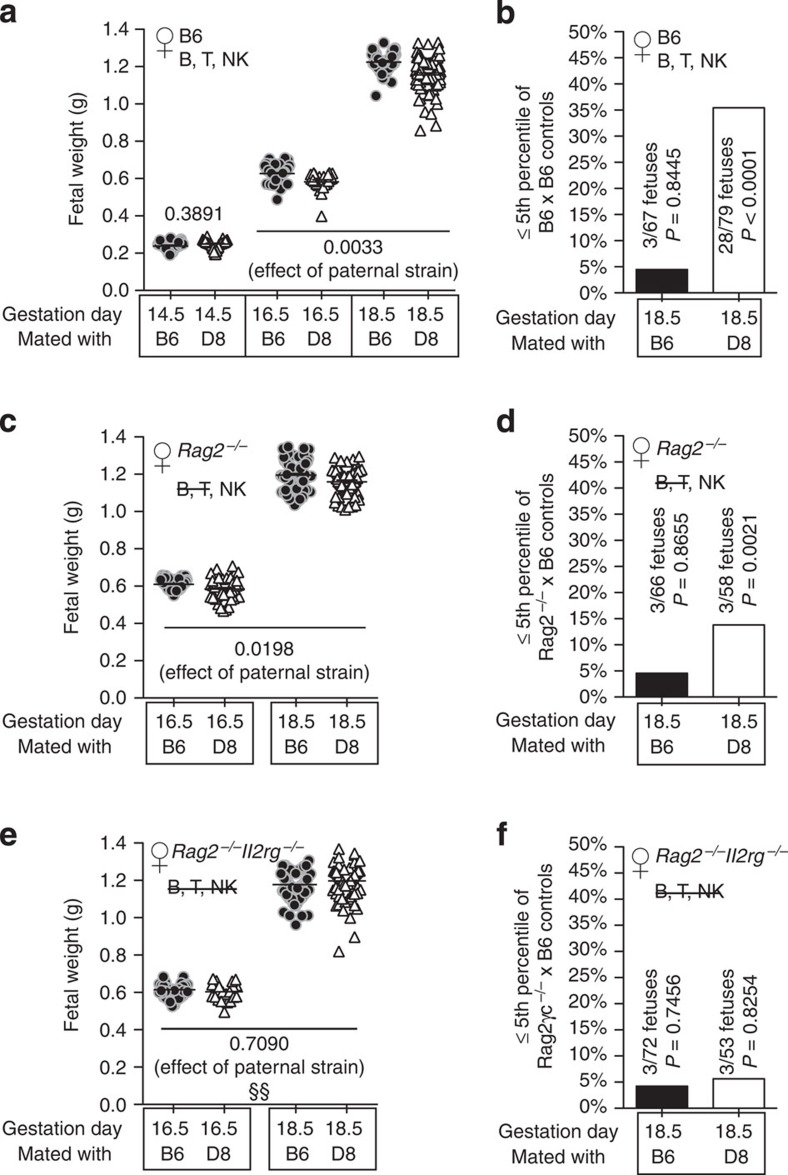
Reduced fetal growth. Fetal weight (**a**,**c**,**e**) in conceptuses from wild-type females (**a**), *Rag2*^−/−^ females lacking B and T cells (**c**) and *Rag2*^−/−^*Il2rg*^−/−^females lacking B, T and NK cells (**e**). *P*-values for the effect of parental cross using a mixed model approach (taking into account inter-litter variability and gestational age), §§*P*<0.005 compared with B6 × B6 matings. Means±s.e.m. Data representative of 3–7 (gd14.5 and 16.5) or 10–11 litters (gd18.5) per cross. (**b**,**d**,**f**) Fraction of fetuses in or below the bottom 5% rank of controls (that is, ≤5^th^ percentile) from wild-type females (**b**), *Rag2*^−*/−*^ females lacking B and T cells (**d**) and *Rag2*^−/−^*Il2rg*^−/−^females lacking B, T and NK cells (**f**). *P-*value from a χ^2^ test comparing the observed fraction with the expected value of 5%. B6.*Rag2*^−/−^*Il2rg*^−/−^females (**e**) carried smaller fetuses than control wild-type B6 females mated with B6 (*P*<0.01 in a mixed model analysis).

Fetal growth and birth weight are critical determinants for reproductive fitness^33^. To assess the impact of excessive NK inhibition on reproductive success, we measured feto-placental growth one day after the completion of arterial remodelling at gd14.5 (ref. 18)^18^, at gd16.5, and immediately before term at gd18.5, to exclude milk uptake as a confounding factor. From gd16.5 onwards, mean fetal weights in B6 × D8 matings were reduced by 6% at gd16.5 and by 9% at gd18.5 (Fig. 8a) compared with B6 × B6 controls. Notably, the overall distribution of fetal weights was disparate, with a >7-fold increase of fetuses within the bottom 5^th^ percentile in B6 × D8 matings compared with B6 × B6 matings (that is, fetuses below the threshold weight for the bottom 5^th^ percentile of control matings were more than seven times more frequent) (Fig. 8b). Placental weight, litter size and number of resorptions remained similar between the crosses throughout gestation (Supplementary Fig. 6). Fetal growth was also lower in D8-mated B6.*Rag2*^−/−^compared with B6-mated B6.*Rag2*^−/−^females, demonstrating that NK cells, but not T or B cells, are responsible for lower birth weight in an MHC dependent manner (Fig. 8c,d), although the absence of *Rag2*-dependent cells did quantitatively modify the phenotype, suggesting that adaptive lymphocytes might contribute to it. Alymphoid B6.*Rag2*^−/−^*Il2rg*^−/−^females^29^ carried smaller fetuses than wild-type B6 mice (*P*<0.01, mixed model analysis) (Fig. 8e), but no specific effect of paternal MHC was seen in these females (*P*=0.709, mixed model analysis), further underscoring the importance of NK cells (Fig. 8e,f). Together, these experiments demonstrate that the presence of paternal H-2D^d^ on trophoblast increased the proportion of fetuses with fetal growth restriction and this depends on uNK-MHC interactions.

### Inhibition of uNK by maternal MHC is equally detrimental

**Figure 9:**
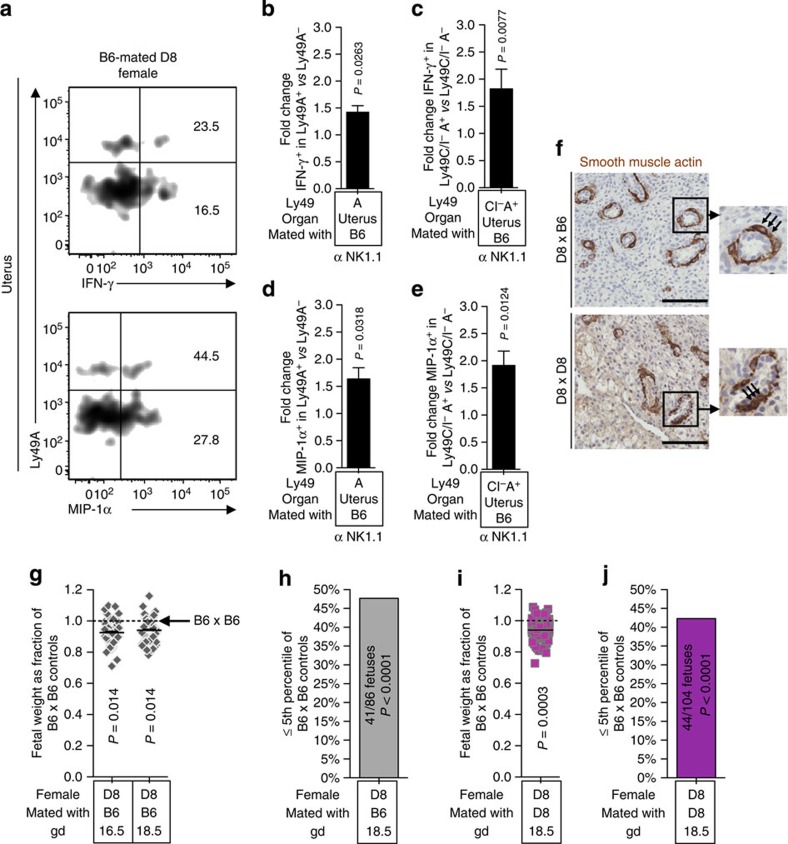
Maternally derived H-2D^d^ also impedes fetal growth and vascular remodelling. (**a**) Density plots showing the proportion of IFN-γ^+^ or MIP-1α^+^ uNK among cells positive or negative for Ly49A. (**b**–**e**) Fold-change in IFN-γ- and MIP-1α-producing cells among uNK expressing Ly49A compared with cells that do not. (**b**) Proportion of IFN-γ^+^ cells among cells expressing Ly49A compared with Ly49A^−^uNK (irrespective of expression of other Ly49). (**c**) Proportion of IFN-γ^+^ cells among cells expressing Ly49A compared with Ly49A^−^uNK (after exclusion of Ly49C/I^+^ cells). (**d**) Proportion of MIP-1α^+^ cells among cells expressing Ly49A compared with Ly49A^−^uNK (irrespective of expression of other Ly49). (**e**) Proportion of MIP-1α^+^ cells among cells expressing Ly49A compared with Ly49A^−^uNK (after exclusion of Ly49C/I^+^ cells). Pooled data from four experiments, *n*=6 mice. *P*-values comparing uNK subset expressing a given Ly49 with those that do not within each mouse using paired Student’s *t*-tests. Means±s.e.m. (**f**) Presence of smooth muscle actin around spiral arteries. Scale bar=100 μm. (**g**–**j**) Fetal growth is reduced in D8 females mated with either B6 (**g**) or D8 (**i**) males. Weights expressed as fraction of fetal weight in B6 × B6 controls. *P*-values for the effect of parental cross using a mixed model approach (taking into account inter-litter variability). Proportion of fetuses in or below the bottom 5% rank of controls (that is, ≤5^th^ percentile) for D8 females mated with either B6 (**h**) or D8 (**j**) males. *P-*value from a χ^2^ test comparing the observed fraction with the expected value of 5%.

Our data demonstrate that uNK in B6 females are not educated by paternal H-2D^d^ but are inhibited by it, resulting in reduced IFN-γ production in response to IL-12 and IL-15, impaired vascular remodelling and fetal growth restriction. To investigate whether the strongly inhibitory nature of the interactions between NKR and MHC was affected by the parental origin of the MHC and/or by the compatibility of the parental MHC, we studied matings where the extra inhibitory MHC class I is expressed only by the mother (D8 × B6 mating) or by both parents (D8 × D8) (Supplementary Fig. 7). In contrast to the mismatched B6 × D8 mating, the contribution of fetal H-2D^d^ in mismatched D8 × B6 matings cannot be resolved because both mother and fetus co-express H-2^b^ and H-2D^d^ (Supplementary Fig. 7). However, in the D8 × B6 mating, one can analyse the impact of H-2D^d^ on both education and inhibition of maternal NK cells. H-2D^d^ educates Ly49A^+^ peripheral NK cells to acquire functional competence but it also inhibits their effector function^4^,^34^. We found that uNK in D8 females are indeed educated through Ly49A (Fig. 9a–e) as reported for peripheral NK in other H-2D^d^-bearing strains^4^. We then evaluated whether Ly49A^+^ NK cell education by H-2D^d^ in transgenic D8 females before pregnancy gives these females an advantage over the B6 females, in which Ly49A^+^ cells are ‘uneducated’. Despite the increased functional competence of Ly49A^+^ uNK in D8 females (Fig. 9a–e), both vascular remodelling (Fig. 9f) and fetal growth (Fig. 9g–h) were reduced, demonstrating that excessive inhibition induced by either parental MHC is detrimental and suggesting that education of maternal uNK through Ly49A is not protective against the complications of pregnancy observed in matings where H-2D^d^ is paternally inherited. Similar pregnancy complications were also observed in D8 × D8 matings, suggesting that strong inhibition of uNK by H-2D^d^ impedes vascular remodelling (Fig. 9f) and fetal growth (Fig. 9i–j) also when parental MHC are matched.

## Discussion

The maternal-fetal interface is the boundary where two genetically different individuals come into close contact. We show here that, in the presence of an MHC known to be a high-affinity ligand for inhibitory NKR, uNK are strongly inhibited. The functional effect is defective remodelling of decidual spiral arteries and compromised fetal growth. Maternal immune responses to fetal antigens, including paternally inherited MHC antigens, do occur, but these are not specifically directed against trophoblast^35^. Our results suggest that uNK respond to MHC molecules expressed on the trophoblast, and these responses have downstream consequences on reproductive fitness. In this work, we unambiguously demonstrate the effects of paternally inherited, mismatched MHC expressed by the trophoblast. Notably, we also show that high affinity engagement of inhibitory NKR by maternally derived MHC has a similarly detrimental effect, and these effects are observed also when parents are matched for the inhibitory MHC.

We have previously shown an association of fetal growth and decidual vascular remodelling with both MHC-linked genes and NKR in mice^8^, but the identity of these genes and how NKR were involved were not clear. This is the first study addressing the functional responsiveness of uNK Ly49 subsets. Here we have tested the effect on uNK activation of a single, paternally inherited MHC class I allotype that engages inhibitory NKR on uNK. Fewer uNK with paternal MHC specificity produced IFN-γ when the strongly inhibitory MHC molecule was present. This impact on NK responsiveness was most pronounced in Ly49C/I^−^Ly49A^+^ uNK cells, but also seen in Ly49A^+^ and Ly49G2^+^ cells (irrespective of expression of other Ly49). No impact on responsiveness was observed for activating Ly49D. Ly49A has the highest affinity for H-2D^d^, whereas Ly49G2^+^ and Ly49D^+^ have very low affinity for H-2D^d^ (refs 24, 25).

MHC-dependent uNK inhibition may be well sustained from when uNK appear in significant numbers (gd6.5) to when vascular remodelling is complete (gd12.5), thus the reduction in uNK function may be cumulative and greater than we could detect at any one point in gestation. The production of IFN-γ is one measurable uNK function, but defects in the production of other factors may contribute to the phenotype observed. NK-produced IFN-γ is key in mediating the pregnancy-specific remodelling of the uterine vasculature in mice^12^,^19^,^20^,^21^, but the mechanisms by which NK-derived IFN-γ mediates arterial remodelling are unclear and IFN-γ is not produced in large amounts by human uNK. uNK cells are the predominant source of IFN-γ in the pregnant mouse uterus and require activating cytokines for its production^12^,^22^. We focussed on DBA^−^NKp46^+^ uNK cells because these are the uNK cells that produce IFN-γ (refs 12, 17, 23), but it should be noted that DBA^+^ uNK cells also express Ly49 receptors^36^ and produce angiogenic factors^17^,^31^, and may thus contribute to vascular changes in response to MHC-dependent signals. VEGF is produced by several cell types, including the uNK in the decidua and those in the MLAp, which surround the arteries supplying the feto-placental unit in rodents^30^. We confirmed that the MLAp stains positively for VEGF, and it is likely that the significant reduction in MLAp volume in matings where uNK were inhibited negatively impacts on VEGF production in this region, with possible negative consequences on decidual vascular changes. The development of the MLAp depends on uNK^12^,^37^, and our data suggest that MHC-dependent uNK inhibition might affect several uterine cells and functions.

F1 offspring of two different inbred strains usually display greater fitness, presumably because of advantageous heterozygosity at a large number of loci, a phenomenon known as ‘hybrid vigour’^38^. With our mating strategy, we are able to pinpoint the effect of a difference in one single MHC locus, which may explain why spiral artery remodelling in B6 × BALB/c matings is not impaired, even though H-2D^d^ is also expressed at the maternal-fetal interface in this mating combination^8^. In the (B6 female × D8 male) mating, the transgenic MHC can affect uNK cells only when they contact placental cells during pregnancy. The outcome is straightforward: this extra, paternally inherited MHC with high affinity for inhibitory NKR does not educate, but instead inhibits uNK and impedes fetal growth and arterial remodelling. These complications happen also when the transgene is expressed either by only the mother in D8 × B6 matings or by both parents in D8 × D8 matings, even if in these matings NK cells develop in the presence of H-2D^d^ and are therefore both tolerant of and educated by H-2D^d^. Further progress in this area awaits deciphering the molecular mechanisms of NK cell education.

In human pregnancies, reduced trophoblast invasion is associated with compromised uterine vascular changes^2^. Using immunohistochemistry, we did not detect changes in the depth of invasion that depended on paternal MHC, although NKR–MHC interactions had a profound effect on arterial remodelling. Data from NK-depleted rats suggest that NK cells may indeed be involved in controlling the degree of trophoblast invasion in rodents^32^. Trophoblast invasion is shallower in normal mouse pregnancies^15^,^39^, and it may be challenging to assess subtle changes in invasiveness in the mouse.

T cells, both effector and regulatory, are found in the human decidua^40^ and have a profound impact on the outcome of allogeneic matings in mice^41^. Since T cells recognize and respond vigorously to allo-MHC molecules^40^,^42^, we performed experiments to determine the contribution of T cells to the phenotype observed in our mating combination, as the extra MHC of transgenic males is allogeneic. We found that T cell-deficient females also exhibited defective fetal growth, suggesting that uNK cells are sufficient to mediate the MHC-dependent effect and that potential T cell responses to the allogeneic MHC are not detrimental. However, fetal growth restriction was milder in T cell-deficient mice, thus T cells may modulate uNK allo-recognition. Indeed, regulatory T cells may dampen uNK cell responses, but the absence of regulatory T cells in T cell-deficient females may alleviate MHC-dependent uNK inhibition.

The impact of KIR-HLA interactions on immune responses depends on the nature of the NKR, which can be either inhibitory or activating^3^. Under physiological conditions, NK cell responsiveness is attuned to the (constant) MHC environment^43^. Changes in the range of MHC molecules available to NK cells can disrupt this balance. Thus, in allogeneic bone marrow transplantations, engagement of an excess of inhibitory KIR on donor NK by host HLA prevents donor cells from clearing the cancer and ultimately impacts negatively on survival^44^. Our results provide strong support for the role of NKR-MHC interactions in determining the outcome of human pregnancy, where decreased utero-placental blood flow leads to complications of pregnancy^2^,^45^.

Low birth weight and fetal programming *in utero* underpin many adult chronic diseases, including cardiovascular and metabolic disease^18^. Applied to human pregnancy, our data may explain the increased likelihood of women with a *KIR AA* genotype carrying a fetus with HLA-C2 to have pregnancy-related complications^7^,^10^,^11^. In the maternal KIR *AA*/fetal *HLA-C2* combination, maternal NK cells will also be strongly inhibited through KIR2DL1 in the absence of activating KIR with HLA-C2 specificity (KIR2DS1). Notably, this combination has the strongest association with an adverse outcome of pregnancy when more copies of *HLA-C2* are found in the fetus than in the mother, in which case, maternal NK cells cannot be attuned or educated to the higher level of fetal HLA-C2 before pregnancy^7^. To this end, we did not find a protective effect of maternal education to paternal H-2D^d^ in our model. This important difference may be due to the exposure time to paternal MHC. While maternal uNK can theoretically interact with trophoblast for months in women, the time window between implantation and completion of remodelling lasts only days in mice.

Our results suggest that strong inhibitory interactions between maternal NKR and MHC can increase the risk of fetal growth restriction, which may also have deleterious downstream consequences on the developmental programming of adult disease^33^. In the long term, our results might raise the possibility of harnessing uNK function by fine-tuning their activation in high-risk pregnancies to prevent certain pregnancy complications. New clinical trials with monoclonal antibodies targeting inhibitory KIR are underway to treat haematological malignancies^46^. Restoring NK cell activity in pregnant women at risk of developing disorders of pregnancy could be a therapeutic strategy worth testing in preclinical models.

## Methods

### Mice

Mouse experiments were approved by the University of Cambridge Ethical Review Panel and carried out in accordance with Home Office Project Licence PPL 80-2347. C57BL/6J (B6; H-2^b^) and BALB/c (H-2^d^) mice were purchased from Charles River UK. D8 mice (H-2D^d^ expressing C57BL/6J transgenic mice) have been described^16^. Expression of the additional MHC gene in these mice is comparable to endogenous H-2D^d^ expression in BALB/c mice. *Rag2*^−/−^and *Rag2*^−/−^*Il2rg*^−/−^mice (both H-2^b^) have been described^28^,^29^. B6*.Rag2*^−/−^mice lack functional B and T lymphocytes, and the remaining immature pro-B and pro-T cells cannot rearrange their antigen receptors and thus fail to achieve antigen specificity. *Rag2*^−/−^*Il2rg*^−/−^mice also lack NK cells due to defective IL-15 signalling. B6 or D8 males were randomly introduced to virgin mice at 7–10 weeks of age, and the timing of conception was determined by detection of a copulation plug representing gd0.5.

### Flow cytometry

Suspensions of splenic or uterine cells were stained for cell surface CD3 (560771 at a 1:100 dilution), CD122 (123210 at a 1:100 dilution), NKp46 (561169 at a 1:100 dilution), DX5 (562453 at a 1:150 dilution), Ly49A (116809 at a 1:100 dilution), Ly49C/I (553277 at a 1:100 dilution), Ly49D (138306 at a 1:300 dilution), Ly49G2 (560730 at a 1:300 dilution), Ly49I (12-5895 at a 1:100 dilution), CD27 (11-0271-81 at a 1:100 dilution), CD11b (101239 at a 1:20 dilution), CD45 (103128 at a 1:200 diltuion), KLRG1 (25-5893-80 at a 1:100 dilution), or intracellular IFN-γ (505822 at a 1:100 dilution) (or MIP-1α (IC450A at a 1:100 dilution)) using commercially available antibodies and cell permeabilization reagents (BioLegend, eBioscience, R&D Systems and BD Pharmingen). Ly49C (4L03311) was a gift from J Laurent. For stimulation, cells were cultured either in the presence of 10 ng ml^−1^ IL-12 and 100 ng ml^−1^ IL-15 for 18.5 h or on plates pre-coated with 10 μg ml^−1^ anti-NK1.1 for 9.5 h (PK136, BioLegend). Brefeldin A (eBioscience, 1 × ) was added for the last 4 h and 1 h after the start of the experiment, respectively. Samples were acquired on an LSRFortessa (BD) and analysed using FlowJo (Treestar).

### Confocal microscopy

Tissue was digested at 37 °C for 0.5 h in the presence of 0.1 WU Liberase DH and 30 μg ml^−1^ DNase (Roche). Cells were stained with PE-conjugated anti-H-2D^d^ antibody (34-5-8S) diluted 1:100, fixed in 2% formalin/PBS for 15 min at 4 degrees Celsius and cytospun onto glass slides^47^. Intracellular staining was performed with polyclonal rabbit anti-cytokeratin (DAKO Z0633 at a 1:100 dilution) followed by AF488-conjugated goat-anti-rabbit at a 1:200 dilution (Invitrogen) and counterstained using DAPI. Images were acquired on a Leica SP5 and analysed using ImageJ.

### Immunohistochemistry

Serial sections of formalin-fixed, paraffin-embedded uterine tissues from gd9.5 pregnant females were cut at 7 μm. Sections at 49 μm intervals were stained with H&E according to standard methods. Cytokeratin was assessed using polyclonal rabbit anti-cytokeratin (DAKO Z0633) at 1:1,000 dilution, following proteinase-K mediated antigen retrieval. Sections for smooth muscle actin staining were subjected to citrate buffer heat-induced epitope retrieval (HIER) and incubated with 1:100 dilution of mouse anti-human smooth muscle actin (DAKO 1A4) according to methods outlined in the mouse on mouse immunodetection kit (Vector Laboratories). VEGF expression was assessed following Tris-EDTA buffer HIER and overnight incubation with 1:500 dilution rabbit polyclonal VEGF-A (Abcam ab46154)^30^. NKp46 expression was visualized in 7 μm thick acetone-fixed cryosections of uterine tissues following incubation with a 1:500 dilution of goat polyclonal anti-NKp46 (R&D Systems, BAF2225) overnight, followed by 1:200 biotinylated horse anti-goat IgG (Vector Laboratories). After colorimetric detection, sections were incubated with 6.6 μg ml^−1^ biotinylated DBA (Vector Laboratories) for a further 30 min. Negative controls were included for immunohistochemical stainings using immunoglobulins or serum matched to the species in which the primary antibodies were raised.

### Stereology

Tissue volumes were determined from serial sections using the Cavalieri method^48^. In brief, cross-sectional areas on serial sections were determined and volumes calculated using the spacing between sections. Arterial remodelling was assessed in the central 2/4 of the decidua basalis on sagittal sections to avoid inclusion of veins that have been shown to be more peripherally located^39^. Three to five implantation sites (corresponding to one uterine horn chosen at random) were analysed per litter. For each implantation site, the five largest vessels were measured in triplicate (three sections 49 μm apart from each other). For all stereological measurements, uterine arteries were tied before dissection to minimize bleeding. Size of lumens and vessels was determined by measuring cross-sectional areas with NDP viewer (Hamamatsu) as this was found to yield more reproducible results than measuring diameters. Differences in size and relative thickness of vessels were confirmed by smooth muscle actin staining. For determination of trophoblast invasion, H&E and cytokeratin stainings were used and analysed as described before^8^ using the most distal section from the fetus that contained fetal red blood cells as a marker for the base of the ectoplacental cone and the most distal section positive for cytokeratin staining as its apex.

### Statistics

Data were analysed using unpaired (separate mice) or paired (cell populations within the same mouse) one-tailed Student’s *t*-tests. Fetal and placental weights were analysed using a mixed model approach to test the effect of parental cross and account for both gestational age and clustering of observations by litter^14^. *P*<0.05 was taken as statistically significant for all tests. Categorical data were analysed using χ^2^ tests to compare observed and expected values. Analyses were performed using GraphPad Prism and IBM SPSS.

## Additional information

**How to cite this article:** Kieckbusch, J. *et al.* MHC-dependent inhibition of uterine NK cells impedes fetal growth and decidual vascular remodelling. *Nat. Commun.* 5:3359 doi: 10.1038/ncomms4359 (2014).

## Supplementary Material

Supplementary Information Supplementary Figures 1-7
