# Corrigendum: MHC-dependent inhibition of uterine NK cells impedes fetal growth and decidual vascular remodeling

**DOI:** 10.1038/ncomms15444

**Published:** 2017-04-20

**Authors:** Jens Kieckbusch, Louise M. Gaynor, Ashley Moffett, Francesco Colucci

Nature Communications 5: Article number: 3359; DOI: 10.1038/ncomms4359 (2014); Published: 02 28 2014; Updated: 04 20 2017

This Article was originally published under a CC BY-NC-ND 3.0 license, but has now been made available under a CC BY 4.0 license. The PDF and HTML versions of the paper have been modified accordingly.

