# Supplementary material for: MHC-dependent inhibition of uterine NK cells impedes fetal growth and decidual vascular remodelling
Source: Nat Commun. 2014 Feb 28;5:3359. doi: 10.1038/ncomms4359 (PMC3948146; doi:10.1038/ncomms4359)
Supplement: Supplementary Information — Supplementary Figures 1-7 [file ncomms4359-s1.pdf]

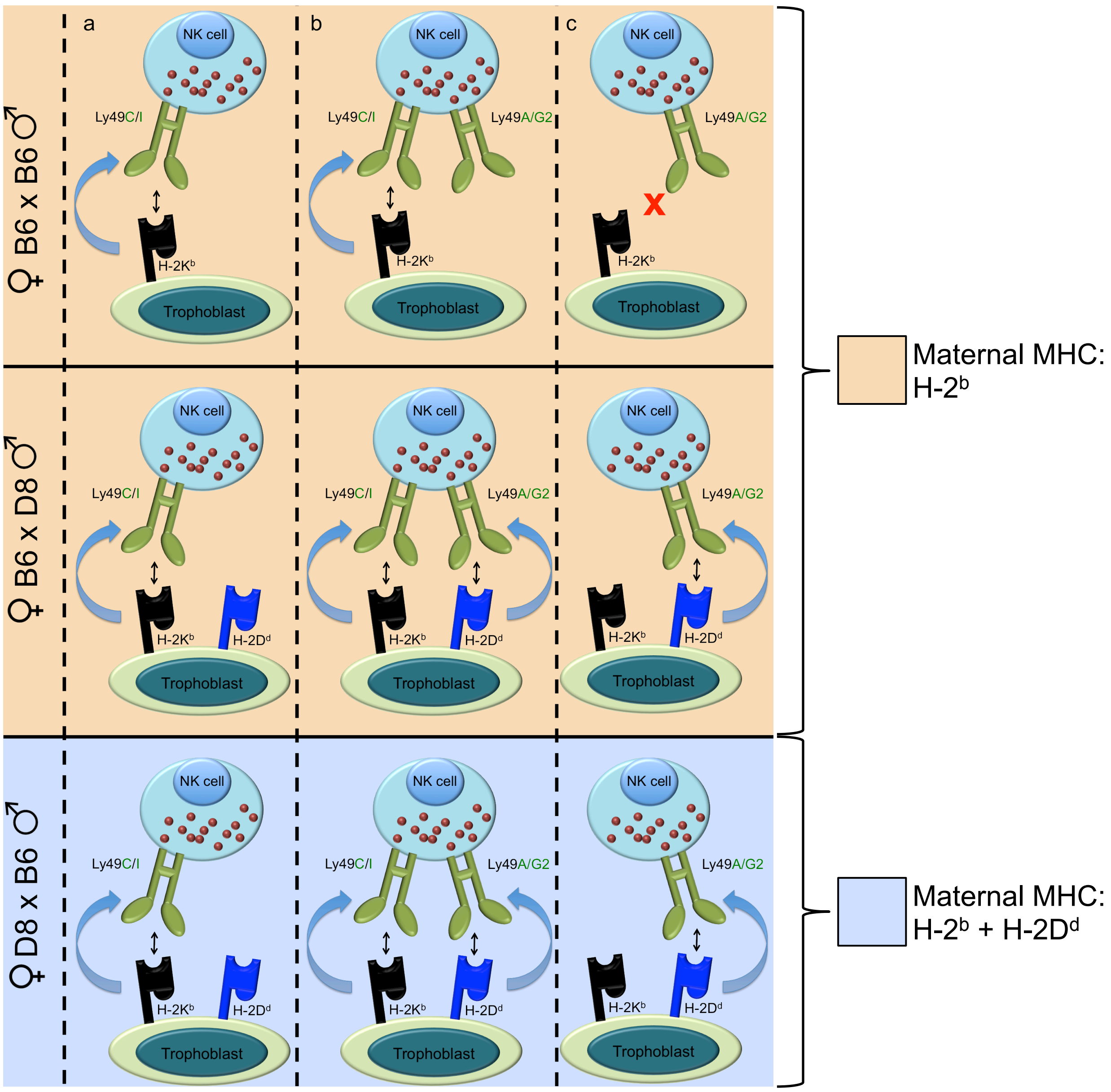

Supplementary Figure 1. MHC-NKR interactions at the maternal-fetal interface. Depicted in columns are cells expressing H-2K^b binding Ly49C and/or Ly49I (**a**), co-expressing Ly49C and/or Ly49I as well as H-2D^d binding Ly49A and/or Ly49G2 (**b**) or expressing Ly49A and/or Ly49G2 (**c**). Depending on the mating combination (top, middle and bottom rows), these different NK subsets can recognise and bind to different MHC class I molecules on maternal tissues (i.e. uterine leukocytes or stroma) and fetal trophoblast cells. These NK subsets will thus potentially be inhibited (blue arrows) through these interactions by different degrees. The mating combinations also differ in the maternal MHC environment (background colour) such that in the top two cases, NK cells develop in the presence of H-2^b prior to pregnancy (grey) or, in the bottom row, in the presence of both H-2^b and transgenic H-2D^d (blue).

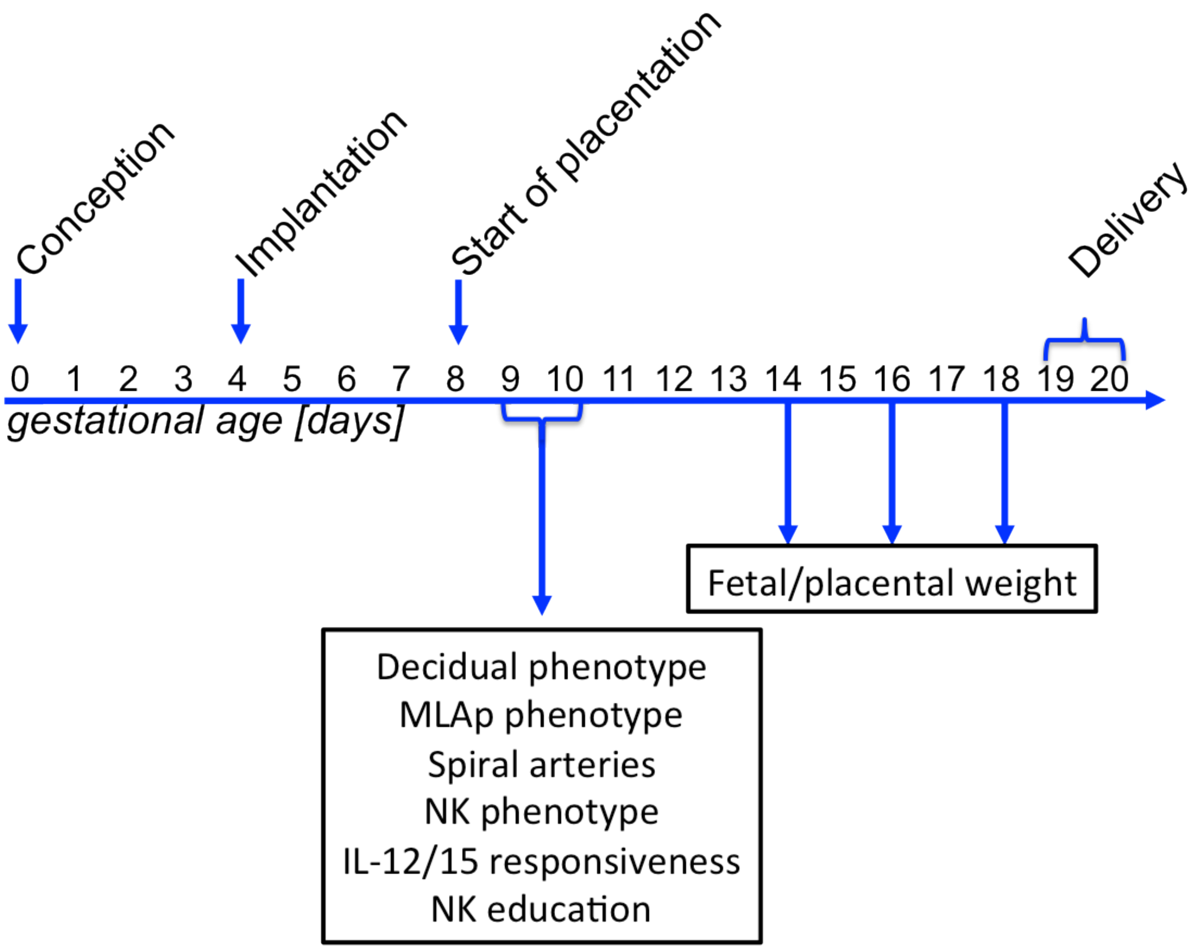

Supplementary Figure 2. Overview and timing of experimental procedures
Defining the morning after detection of a copulation plug as gd0.5, mouse implantation occurs at around gd4.5 and placentation (i.e. the formation of the placenta) commences at around gd8.5. In this study, most parameters of pregnancy were assessed around mid-gestation (gd9.5 and 10.5) whereas fetal and placental weights were used as primary outcomes toward the end of gestation but before delivery to avoid confounding effects through milk uptake. MLAp = Mesometrial Lymphoid Aggregate of pregnancy

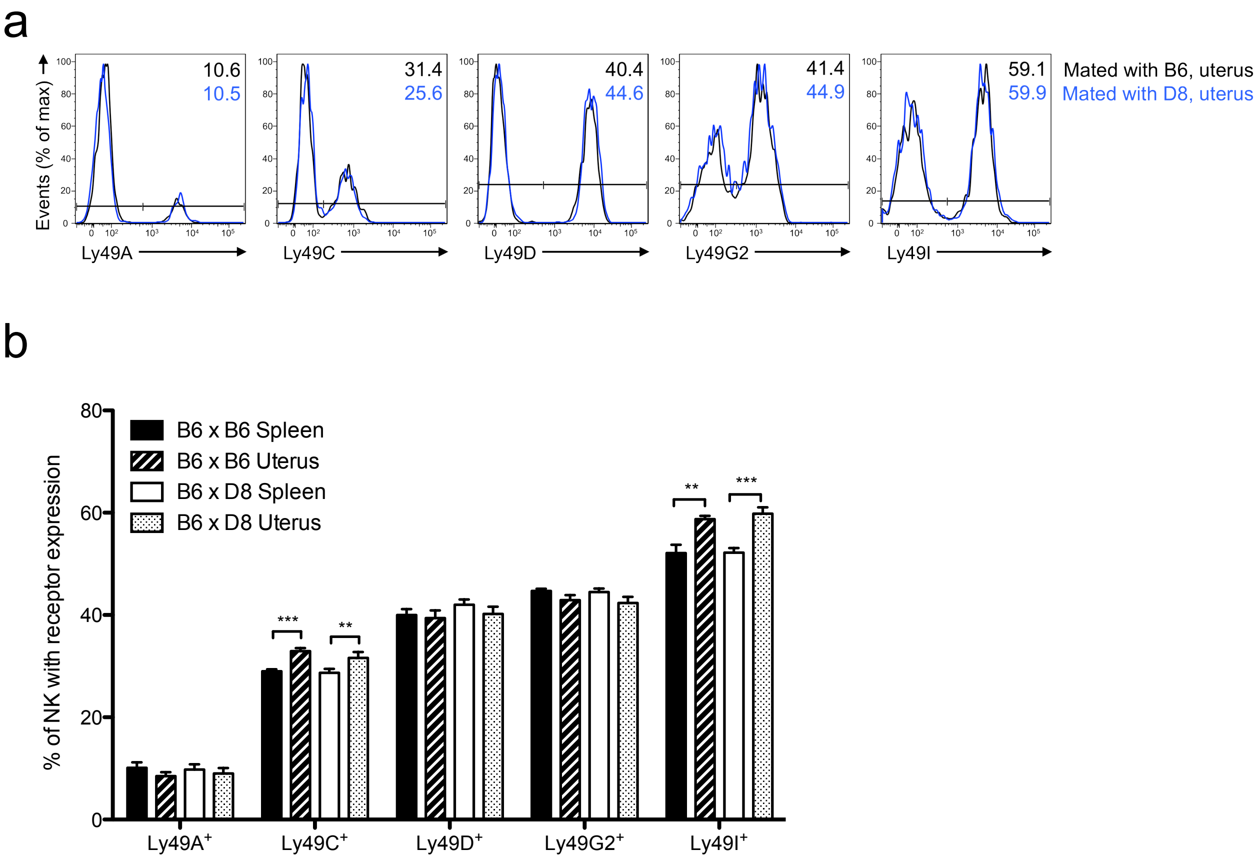

Supplementary Figure 3. The uNK Ly49 repertoire is skewed towards recognition of maternal MHC

a, Representative histograms showing Ly49 repertoire of $CD3^-$, $CD122^+$, $NKp46^+$ cells in spleen and uterus (gd10.5). **b**, Frequency of NK cells expressing a given Ly49 receptor (irrespective of expression of other Ly49). Pooled data from 6 independent experiments, n = 4 – 6 (Ly49I) or n = 8 – 11 (all other Ly49). **, $p < 0.01$; ***, $p < 0.0005$ from paired Student's t-tests. Means ± SEM.

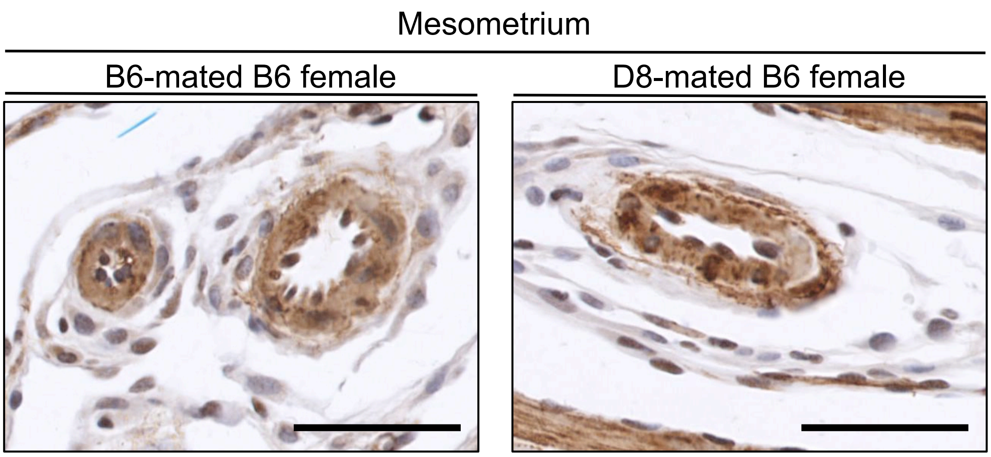

Supplementary Figure 4. Mesometrial arteries distant from the NK-rich decidua remain unremodelled

Smooth muscle actin staining of mesometrial areas on sections from gd9.5 pregnant females. Bar = 50 µm

a

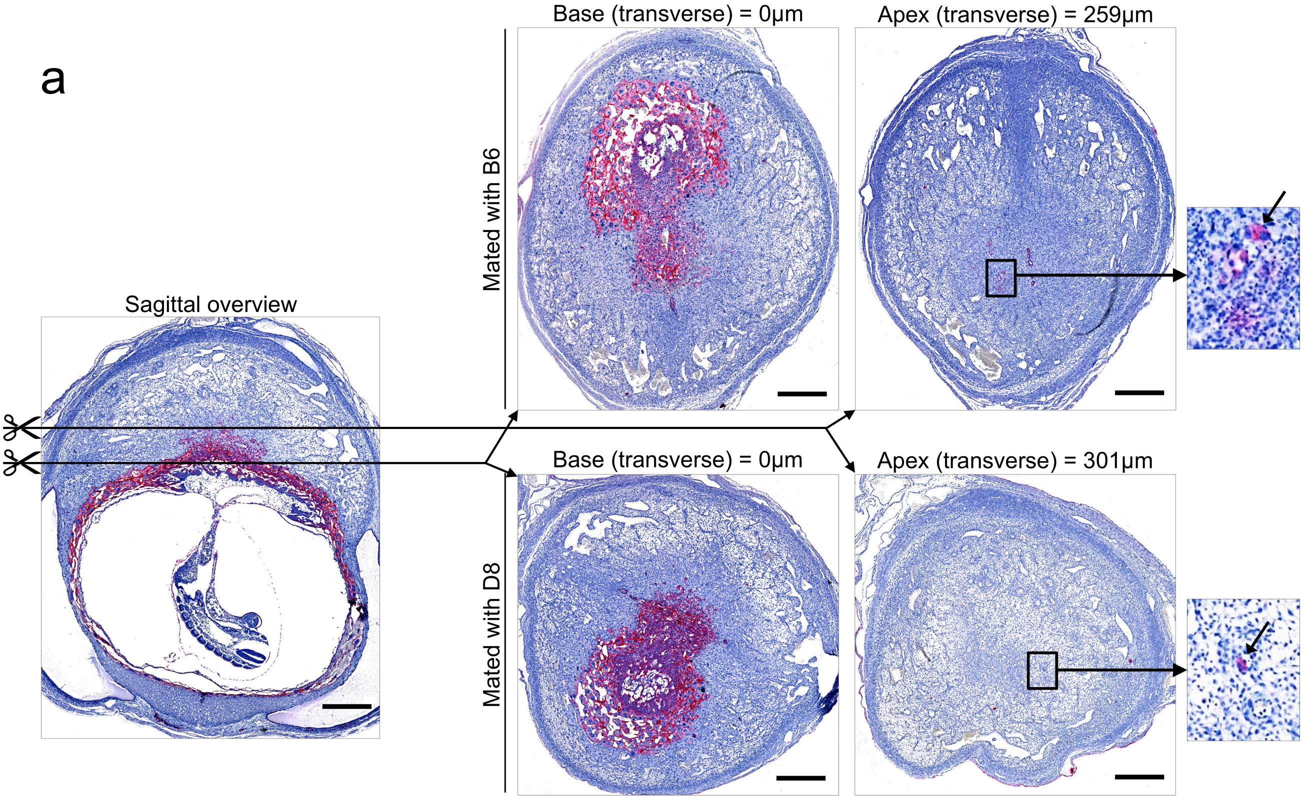

b

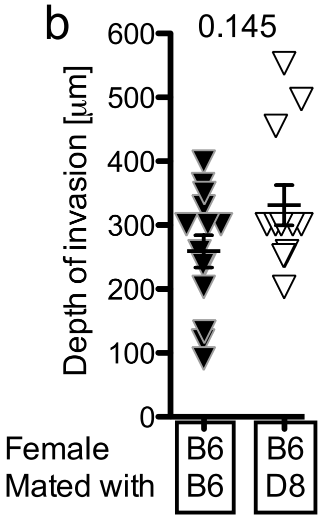

Supplementary Figure 5. Inhibition of uNK cells does not affect invasion of trophoblast cells

a, Cytokeratin staining illustrating the extent of trophoblast invasion. Sagittal cross-section (left) of a gd9.5 implantation site showing how transverse sections (lines) through the implanting placenta were obtained. Base (middle panel, invasion depth 0µm) defined as the most distal section from the fetus containing both trophoblast and fetal red blood cells. Apex (right panel) defined as the most distal slide containing trophoblast cells and used to quantify trophoblast invasion. Bar = 500µm. **b**, Quantification of depth of trophoblast invasion. Means ± SEM, representative of uterine horns from 4 litters per cross (n= 12-14 implantation sites). *P*-value from unpaired Student's t-test.

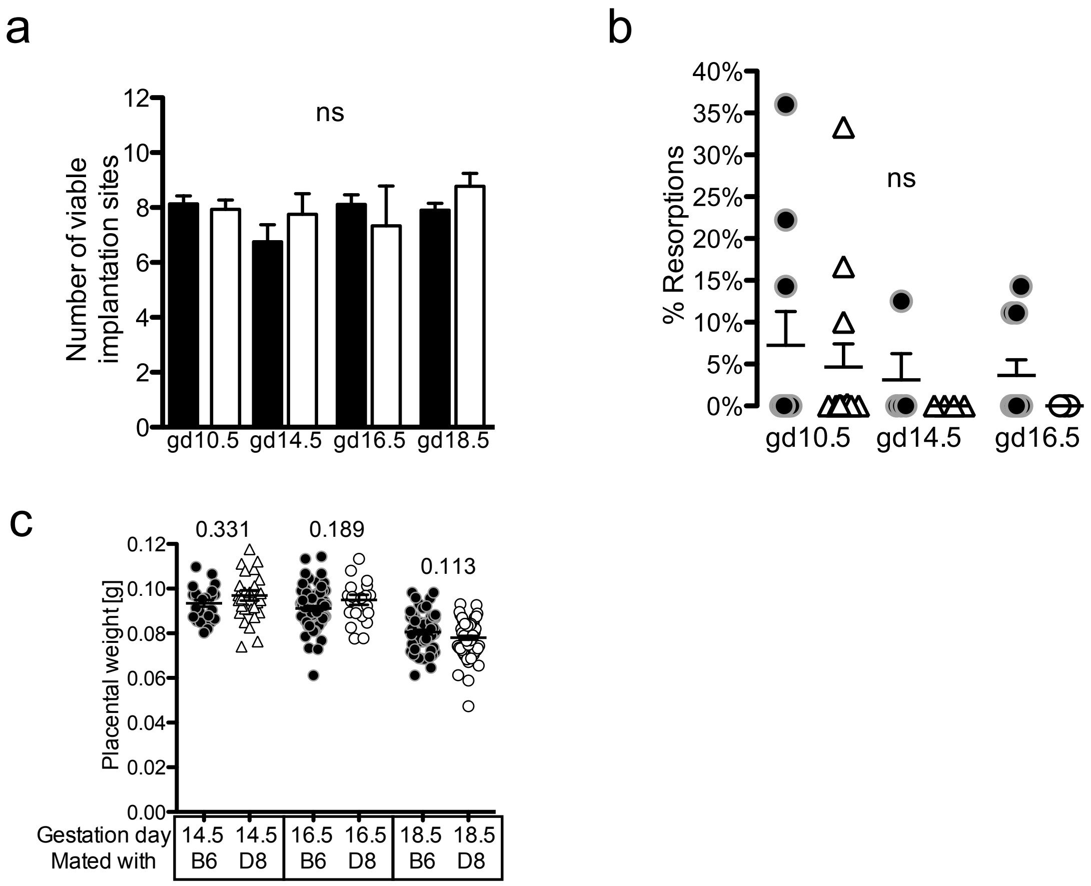

Supplementary Figure 6. Litter size and placental growth are unaffected by paternal H-2D^d expression on trophoblast

a, Number of viable implantation sites across gestation. **b**, Fraction of fetal resorptions observed across gestation. **c**, Placental weight across gestation. Black symbols/bars: B6 females mated with B6 males, white symbols/bars: B6 females mated with D8 males. Means ± SEM. Data representative of 6-11 litters. *P*-values from unpaired Student's t-test (**a+b**) and mixed model approach (**c**) taking inter-litter variability into account.

	No H-2D^{d}	H-2D^{d} in father	H-2D^{d} in mother	H-2D^{d} in both
**Mating**	B6 x B6	B6 x D8	D8 x B6	D8 x D8
♀	b	b	b + d	b + d
♂	b	b + d	b	b + d
	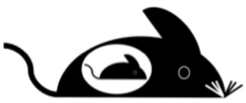	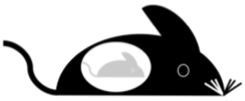		
**Placenta**	b	b + d	b + d	b + d
**Arterial remodelling**	✔	↓	↓	↓
**Fetal growth**	✔	↓	↓	↓

Supplementary Figure 7. Mating combinations used and summary of findings

This cartoon summarises our main finding that strongly inhibitory MHC-NKR interactions are detrimental for fetal growth and arterial remodelling, regardless of the parental origin of the inhibitory MHC and irrespective of parental MHC matching. The outcome of the mating combinations were always compared to syngeneic B6 x B6 matings, in which the strongly inhibitory H-2D^{d} is not expressed. Notably, all mice are B6, including the D8 transgenics.

Paternal expression of inhibitory MHC. Maternal uNK cells can encounter and interact with paternally inherited MHC class I molecules only during pregnancy. Therefore, the B6 x D8 mating combination is the only one in which the effect of the paternally inherited H-2D^{d} expressed by the trophoblast (placenta) can be unambiguously determined. The paternally inherited MHC with high affinity for inhibitory NKR does not educate but inhibits a large fraction of maternal uNK (H-2D^{d} inhibits Ly49A and Ly49G2) and impedes arterial remodelling and fetal growth.

Maternal expression of inhibitory MHC. In these two mating combinations, uNK cells encounter and interact with H-2D^{d} throughout their development. Exposure of maternal NK cells to self MHC prior to pregnancy educate them to be tolerant of that MHC and to mature and acquire functional competence. This however does not seem to counter the inhibitory effect of H-2D^{d} on uNK, which impedes arterial remodelling and fetal growth irrespective of whether parental MHC are mismatched (D8 x B6) or not (D8 x D8).
